# Gender and Sexual Orientation Differences in Human Papillomavirus (HPV) Vaccine Uptake among Chinese Young Adults

**DOI:** 10.3390/ijerph15061099

**Published:** 2018-05-28

**Authors:** Edmond Pui Hang Choi, Janet Yuen Ha Wong, Angel Yin Yim Lau, Daniel Yee Tak Fong

**Affiliations:** School of Nursing, LKS Faculty of Medicine, The University of Hong Kong, Hong Kong, China; janetyh@hku.hk (J.Y.H.W.); yylau11@hku.hk (A.Y.Y.L.); dytfong@hku.hk (D.Y.T.F.)

**Keywords:** human papillomavirus, vaccine, vaccine uptake, Chinese

## Abstract

To date, empirical studies on HPV vaccine uptake are still limited in Chinese populations and mainly conducted in female cohorts. In order to inform health services planning and health promotion programmes for HPV vaccination, this cross-sectional study aimed to report the prevalence of self-reported HPV vaccination status and to examine gender and sexual orientation differences in the uptake of HPV vaccine in Chinese college students. The overall prevalence of HPV vaccine uptake was 27.6% (*n* = 242), with a significantly higher prevalence in females (39.7%) than in males (4.7%). 91.4% of subjects heard about HPV vaccination, with a significantly higher prevalence in females (93.8%) than in males (86.8%). The prevalence of HPV vaccine uptake was only 2.6% for bisexual/ homosexual males and 5.0% for heterosexual males. Only 45.8% of the overall subjects knew HPV vaccination is not for females only, with a significantly higher prevalence in females (49.7%) than in males (38.6%). The low prevalence of male HPV vaccine uptake and awareness called for the need to have more male-specific HPV campaigns to promote HPV vaccination awareness and uptake in males to reduce the overall prevalence of HPV infection.

## 1. Introduction

Genital human papillomavirus (HPV) infection is the most common sexually transmitted infection (STI) in the world [1]. Transmission of genital HPV infection is mainly through sexual contact [2]. Up to 75% of all sexually active people would have HPV infection at some point during their lifetime although most infections are subclinical [3]. The health impact of HPV infection on women’s health is substantial, with 70% of cervical cancers being attributable to HPV type -16 and -18. A meta-analysis conducted in 2007 found that among Asian women with invasive cervical cancer, HPV-16 was the predominant type (52.4%), followed by HPV-18 (14.5%) [4]. Cervical cancer is the second most common cancer in women in less developed countries [5]. In Hong Kong, cervical cancer was the 8^th^ commonest cancer among females and accounted for 3.3% of all new cancer cases in females in 2014 [6]. There is also evidence linking HPV infection with cancers of the anus, vulva, vagina and penis [7]. The economic burden of HPV infection is substantial. It was estimated that the lifetime total medical cost of HPV infection for men and women aged 15 to 24 is US$2.9 billion, which makes HPV the second most expensive STI after human immunodeficiency virus [8].

### 1.1. HPV Vaccination in Asian Settings

A systematic review conducted in 2010 on HPV vaccine awareness and acceptance among women in the Asia Pacific region found a large variation in HPV vaccine awareness among and within countries [9]. For example, a study on female college students in Malaysia found that only 10.3% of participants had heard of HPV vaccine [10] while a study in Australia found that 83% of the surveyed women had heard of it [11]. Besides, a study on university students in mainland China in 2013 found that only 5.4% of students had heard of HPV vaccine (7.0% in males and 3.9% in females); and only 70.6% of students (71.8% in males and 69.4% in females) were willing to take HPV vaccines [12]. The systematic review also found a large intra-country variation in Australia (ranging from 33% in the study by Giles and Garland [13] to 83% by Weisberg and colleagues [11]). The variations might be due to the year of data collection relative to the year of the countries’ approval of the vaccine and the differences in study populations [9]. In Australia, the HPV vaccine was introduced in 2007 through the National Immunisation Program [14]. Therefore, Giles and Garland’s study, which was conducted in 2006, might find a relative low awareness of HPV vaccine [13] while Weisberg and colleagues’ study, which was conducted in 2008, might find a higher awareness of HPV vaccine [11]. It is noteworthy that the review could only identify one study which assessed the actual vaccine uptake in the region and the study found 56% of young Australian women had received HPV vaccination [11].There is a need to have more epidemiological studies to estimate the awareness and uptake of HPV vaccine for the development of HPV promotions and health services planning in Asia.

Besides, cultural beliefs and social sigma might also affect the HPV vaccine uptake in Asia. A recent qualitative study on Chinese college students identified several concerns about HPV vaccination [15]. First, study subjects disagreed on the recommended vaccination age of 9 to 26 years, stating that Chinese youths start to engage in sexual behaviour later than Western people. Second, some participants thought that the vaccine was developed in western countries. Therefore, it is not suitable for Chinese. Another qualitative study in Malaysia found the some women were concerned about the potential to be perceived as promiscuous and sexual active [16]. It was possible that misconceptions and concerns about HPV vaccine would affect the willingness of HPV vaccine uptake in Asian populations. Therefore, the estimated prevalence of HPV vaccine uptake in Western populations might not be transferable to Asian populations.

### 1.2. HPV Vaccination in Hong Kong

Currently, there are three registered HPV vaccines in Hong Kong. 4-valent Gardasil was licensed in Hong Kong in 2006; 2-valent Cervarix in 2008; and 9-valent Gardasil in 2015. Of these, 4-valent Gardasil and 9-valent Gardasil are approved for use in both males and females. Unlike some Western countries such as Australia [17], HPV vaccination is not included in the immunisation programme in Hong Kong. Each injection will cost on average HK $1500 (US $192), and therefore the complete vaccination sequence of three injections will cost around $4500 (US $576). In 2008, a study on girls aged 11 to 18 years in Hong Kong reported that 40.3% of them had heard of HPV vaccines but only 2.4% of them had received HPV vaccination [18]. There were some possible explanations for the low HPV vaccine uptake rate in the study. First, the study was conducted in 2008 when HPV vaccines were still new to people in Hong Kong. Second, HPV was not part of the immunisation programme in Hong Kong. Besides, despite the approval and effectiveness of HPV vaccination in males especially for men who have sex with men (MSM), health promotion on HPV vaccination in Hong Kong is predominately developed for females. As males are the primary transmission vector of HPV to females, concurrent efforts to vaccinate both males and females can provide a more effective way to reduce the prevalence of HPV infections in the population, leading to the decrease in the incidence of cervical cancer.

### 1.3. Knowledge Gaps and Needs of the Present Study

To date, empirical studies on HPV vaccine uptake are still limited in Chinese populations. Besides, most Chinese studies about HPV vaccine uptake, and knowledge and beliefs about HPV vaccination were exclusively conducted in females. Furthermore, study findings in mainland China might not be transferable to Hong Kong because HPV vaccines were just approved in mainland China in 2016 and HPV vaccines were gradually available community health centers across 17 provinces in mainland Chinese recently (after ten years of the HPV vaccine being available in developed counties including Hong Kong) [19]. In order to inform health services planning and develop health promotion programmes for HPV vaccination, this study aimed to report the prevalence of HPV vaccination and to examine gender and sexual orientation differences in the uptake of HPV vaccine among Chinese young adults.

## 2. Material and Methods

It was the secondary analysis of a study about the health needs of emerging adults. The study details have been reported elsewhere [20].

### 2.1. Sampling and Setting

Study subjects were recruited in four major universities in Hong Kong by convenience sampling because we had no access to all students in the selected universities. In order to increase the representativeness of the study sample, we included both government-funded and private universities and recruited subjects in different academic terms (from July 2014 to November 2015).

Potential participants were invited to complete a self-administered questionnaire. Questionnaires were available in English and Chinese. Subject were excluded if they refused to join the study or they did not speak or understand Chinese or English.

### 2.2. Outcomes Measures

Subjects were asked (i) if they had heard about HPV vaccination and (ii) if they had had HPV vaccination before. To assess their perception on HPV vaccination, they were asked if HPV vaccination is meant for females only. These questions were responded on the options of “true”, “false” and “unsure”.

### 2.3. Consent and Ethics

Written informed consent of study participants was obtained. The study protocol was approved by the institutional review board of all participating universities (UW 14-290, CREC Ref. No. 2015.186, and H000873). The authors declare no conflicts of interest.

### 2.4. Data Analysis

According to the Centers for Disease Control and Prevention (CDC) Advisory Committee on Immunisation Practices, catch-up vaccination should be strongly encouraged for females aged 13 to 26 and male aged 13 to 21 who were not vaccinated previously or have not completed the 3-dose series. Therefore, only males under age 22 and females under age 27 were included in the data analysis. Chi-square test and simple logistic regression were conducted to examine gender and sexual orientation differences in the prevalence of HPV vaccination respectively.

## 3. Results

888 subjects were eligible to join this study. 65.5% of subjects (*n* = 582) were female. The mean age was 20.1 (standard deviation: 1.5). 4.5% of subjects (*n* = 40) were bisexual or homosexual males while 8.5% of subjects (*n* = 75) were bisexual or homosexual females. 74.9% of subjects (*n* = 665) were born in Hong Kong while 15.5% of subjects (*n* = 138) were born in mainland China.

Table 1 shows the result of gender difference. The overall prevalence of HPV vaccine uptake was 27.6%, with a significantly higher prevalence in females (39.7%) than in males (4.7%). 91.4% of subjects heard about HPV vaccination, with a significantly higher prevalence in females (93.8%) than in males (86.8%). Only 45.8% of subjects knew HPV vaccination is not for females only, with a significantly higher prevalence in females (49.7%) than in males (38.6%).

Table 2 shows the result of sexual orientation difference. In line with the results of gender difference, heterosexual males and bisexual/homosexual males had a lower prevalence of HPV vaccine uptake than the heterosexual females did. The prevalence of HPV vaccine uptake was only 2.6% for bisexual/ homosexual males and 5.0% for heterosexual males. Heterosexual males were less likely to hear about HPV vaccination and to know that HPV vaccination is not for females only than their counterparts.

## 4. Discussion

Before we discuss the study findings and their implications, it is important to note the limitations of the present study and the study findings should be interpreted with caution. First, the subjects were recruited in university setting by convenience sampling. Strictly speaking, the results obtained in the present study can only be applied to the people who completed the survey. However our findings can provide pilot data for the development school-based interventions and health promotion for students in high schools as well as universities. Second, data on HPV vaccine knowledge and attitude were not collected. Further studies should collect those data in order to explore their association with vaccination behaviour. Third, the self-reported vaccination status could be subject to recall bias. However, we believe such recall bias should be low because of the expensive self-purchased HPV vaccine in Hong Kong.

Even though a vast majority of male students (86.8%) had heard of HPV vaccination, we observed a very low overall prevalence of HPV vaccine uptake among males (4.7%), which is much lower than the 22% reported in a US study on men aged 18 to 22 years [21]. Besides, less than half of the overall study sample knew that HPV vaccination is not only for females. Although the estimated prevalence might be biased because of the sampling method, it is worthy of discussing some possible explanations. First, HPV vaccination is not included in the immunisation programme in Hong Kong. It is a self-financed item. Therefore, people in Hong Kong might not be willing to have HPV vaccination because of the costs. Second, public health promotions of HPV vaccination are predominately developed for females in Hong Kong. HPV vaccine marking strategies have labelled HPV as a “woman’s disease” [22]. Moreover, the term “cervical cancer vaccine” is commonly used in health promotion material. Therefore, people might think that HPV is not relevant to males. As suggested by a systematic review, key barriers to HPV vaccination among male adolescents included the lack of perceived benefit or need to vaccinate males, lack of awareness that vaccine can be given to males, not receiving a health care professional’s recommendation for the HPV vaccine and the cost of the vaccine [23]. Furthermore, health care professionals may have a preference of vaccinating females over males and think that male-vaccination is not as cost-effective as female-vaccination.

The low HPV uptake rate in both males and females in Hong Kong can be attributed to the attitude and knowledge of parents because it is usually up to parents to decide whether their child is vaccinated [24] and this decision is often influenced by the knowledge and attitude of parents [25,26]. A multicenter national survey in China reported that only 36.2% of the parents were willing to vaccinate their child [27]. Among reasons against HPV vaccination in children, the most frequently cited was “worry about its safety” (fathers: 62.4%; mothers; 67.9%), followed by “children are too young to have risk of cervical cancer” (fathers: 40.0%; mothers; 44.6%), “worry about its effectiveness” (fathers: 31.8%; mothers: 39.6%) and “it has not been widely used” (fathers: 33.2%; mothers: 39.3%) [27]. The study also found that parental knowledge of HPV was associated with acceptability of HPV vaccination in children [27]. A study in Hong Kong also reported that only 32% of surveyed women agreed to have HPV vaccination for their children [28]. Besides, recommendation of HPV immunisation by healthcare providers has been recognized as an important factor in the individual’s willing ness to receive the vaccine [29]. A study in Hong Kong reported that the HPV-related knowledge level of primary care physicians was low [29]. The study also identified some barriers to advise adolescents aged 10 to 17 years for vaccination. They are “parental refusal due to safety concerns”, “parental reluctance to discuss sexuality and sexually transmitted diseases” and “physicians being regarded as hard selling an expensive vaccine” [29]. A study conducted between 2009 and 2010 in the US found that 77% of healthcare providers favored vaccinating males but only 12% offered HPV vaccination to males because the healthcare providers felt that parents would not be interested in vaccinating sons and were not really aware of the seriousness of HPV-related diseases in males [30].

The prevalence of HPV vaccine uptake among bisexual/homosexual males was way lower than those reported in previous studies. A study in the US found that 21% of men who have sex with men had received ≥1 dose of HPV vaccine [31]. This situation is worrisome because MSM are at particularly high risk for HPV infections due to their sexual practice. A study found that HPV-16 and/or -18 were detected in 37% of MSM [32]. Another study found that MSM were more likely to have anal cancer than non-MSM (odds ratio: 17.3) [33]. There is a need to increase the HPV vaccine uptake among MSM in Chinese populations. In fact, HPV vaccine can also benefit MSM who have prior HPV exposure [34]. First, the vaccine can prevent people from contracting other types of HPV. Second, even though HPV vaccine cannot clear current infection, it might protect people who are previously exposed to HPV against developing an infection at uninfected sites [35,36].

Despite the limitation of the sampling method, the present study can serve the purpose of need assessment which can provide some pilot information for intervention development. Given the low prevalence of HPV vaccination among college students in Hong Kong, there is a need to increase vaccine uptake in this population. There are some possible strategies. First, all students should be screened for HPV vaccination status. For students who do not receive the first dose of HPV vaccine, HPV vaccination decision aid should be provided to them in order to facilitate their decision-making on HPV vaccination. Second, online education materials about HPV infections and HPV vaccination should be readily accessible to college student in order to increase the knowledge and awareness of HPV infections and the vaccines. Third, given the low prevalence of HPV vaccination among male students, especially for MSM, male-specific education materials about HPV infections and HPV vaccination should be developed in order to promote HPV vaccine update among males. A study found that MSM with a recommendation of HPV vaccination were over 40 times more likely to have been vaccinated [31]. Fourth, given that HPV vaccines is approved for use in children from the age of 9, health promotion of HPV vaccine uptake should also start in primary school. Fifth, given that parents and healthcare providers play an important role in making decisions on HPV vaccination, there is a need to increase the HPV-related knowledge and to foster positive attitude towards HPV vaccination among parents and healthcare providers. Last but not least, interventions and programmes to improve parent-child communication about sexuality should be developed because a study found that mother-daughter communication about sex was an important predictor of HPV vaccination among college students.

## 5. Conclusions

This is one of the first studies to explore gender and sexual orientation differences in HPV vaccination and awareness among Chinese populations. We found a low prevalence of HPV vaccine uptake among Chinese young males, especially for MSM; and low awareness of male HPV vaccination. Our study findings urge the need to have more health promotions on male HPV vaccination in the public in order to increase the vaccine uptake rate. It is particularly important to MSM who are more susceptible to HPV infections and related adverse health outcome.

## Figures and Tables

**Table 1:** The prevalence of HPV vaccination and HPV-related knowledge by gender.

Response	All	Male	Female	Chi Square Test
	*N* = 888	*N* = 306	*N* = 582	*p*-Value
Vaccine uptake				
Q1: HPV vaccination, *n* (%), *n* = 876 ^				***<0.001***
Yes	242 (27.6%)	14 (4.7%)	228(39.7%)	
No	573 (65.4%)	260 (86.4%)	313 (54.4%)	
Unsure	61 (7.0%)	27 (9.0%)	34 (5.9%)	
Overall	876	301	575	
Knowledge				
Q2 I have heard about HPV vaccination, n (%), *n* = 879 ^				***0.002***
Yes	803 (91.4%)	263 (86.8%)	540 (93.8%)	
No	33 (3.8%)	19 (6.3%)	14 (2.4%)	
Unsure	43 (4.9%)	21 (6.9%)	22 (3.8%)	
Overall	879	303	576	
Q3: HPV vaccination is for females only, *n* (%), *n* = 879 ^				***0.005***
Yes	290 (33.0%)	109 (36.0%)	181 (31.4%)	
No	403 (45.8%)	117 (38.6%)	286 (49.7%)	
Unsure	186 (21.2%)	77 (25.4%)	109 (18.9%)	
Overall	879	303	576	

Note: ^ 12 subjects did not answer Q1 and 9 subjects did not answer Q2 and Q3.

**Table 2:** The prevalence of HPV vaccination and HPV-related knowledge by sexual orientation.

Response	Heterosexual Male	Bisexual/Homosexual Male	Heterosexual Female	Bisexual/Homosexual Female	Chi Square Test
	*N* = 266	*N* = 40	*N* = 505	*N* = 75	*p*-Value
Vaccine uptake					
Q1: HPV vaccination, *n* (%), *n* = 874 ^					*<0.001*
Yes	13 (5.0%)	1 (2.6%)	196 (39.2%)	30 (41.1%)	
No	225 (85.9%)	35 (89.7%)	273 (54.6%)	40 (54.8%)	
Unsure	24 (9.2%)	3 (7.7%)	31 (6.2%)	3 (4.1%)	
Overall	262	39	500	73	
Knowledge					
Q2: I have heard about HPV vaccination, *n* (%), *n* = 877					*0.017*
Yes	226 (85.9%)	37 (92.5%)	468 (93.4%)	70 (95.9%)	
No	18 (6.8%)	1 (2.5%)	13 (2.6%)	1 (1.4%)	
Unsure	19 (7.2%)	2 (5.0%)	20 (4.0%)	2 (2.7%)	
Overall	263	40	501	73	
Q3: HPV vaccination is for females only, *n* (%), *n* = 877					*0.007*
Yes	89 (33.8%)	20 (50.0%)	154 (30.7%)	27 (37.0%)	
No	102 (38.8%)	15 (37.5%)	250 (49.9%)	34 (46.6%)	
Unsure	72 (27.4%)	5 (12.5%)	97 (19.4%)	12 (16.4%)	
Overall	263	40	501	73	

Note: ^ 2 subject who did not indicate the sexual orientation was exclude in this analysis. 12 subjects did not answer Q1 and 9 subjects did not answer Q2 and Q3.
